# The waterbodies of the halo-volcanic Dallol complex: earth analogs to guide us, where to look for life in the universe

**DOI:** 10.3389/fmicb.2023.1134760

**Published:** 2023-07-14

**Authors:** Hugo Moors, Mieke De Craen, Carla Smolders, Ann Provoost, Natalie Leys

**Affiliations:** ^1^Microbiology Unit, Belgian Nuclear Research Center (SCK CEN), Nuclear Medical Applications Institute (NMA), Mol, Belgium; ^2^Research and Development Disposal, Belgian Nuclear Research Center (SCK CEN), Waste and Disposal (W&D), Mol, Belgium; ^3^European Underground Research Infrastructure for Disposal of Nuclear Waste in Clay Environment, EIG EURIDICE, Mol, Belgium

**Keywords:** Dallol, habitability, chaotropicity, water activity, salinity

## Abstract

Microbes are the Earth life forms that have the highest degree of adaptability to survive, live, or even proliferate in very hostile environments. It is even stated that microbes can cope with any extreme physico-chemical condition and are, therefore, omnipresent all over the Earth: on all the continents, inside its crust and in all its waterbodies. However, our study suggests that there exists areas and even water rich environments on Earth where no life is possible. To support the fact that water rich environments can be lifeless, we performed an extensive survey of 10 different hyper extreme waterbodies of the halo-volcanic Dallol complex (Danakil depression, Ethiopia, Horn of Africa). In our study, we combined physico-chemical analyses, mineralogical investigations, XRD and SEM–EDX analyses, ATP measurements, 16S rDNA microbial community determinations, and microbial culturing techniques. According to our findings, we suggest that the individual physico-chemical parameters, water activity, and kosmo-chaotropicity, are the two most important factors that determine whether an environment is lifeless or capable of hosting specific extreme lifeforms. Besides, waterbodies that contained saturated levels of sodium chloride but at the same time possessed extreme low pH values, appeared to be poly-extreme environments in which no life could be detected. However, we clearly discovered a low diversity microbial community in waterbodies that were fully saturated with sodium chloride and only mildly acidic. Our results can be beneficial to more precisely classify whole or certain areas of planetary bodies, including water rich environments, as either potentially habitable or factual uninhabitable environments.

## 1. Introduction

The question whether extraterrestrial life is or can be present, elsewhere in the universe, arose most likely at the same time when humans realized that besides Earth, other planetary bodies exist in space. The current technology allows space travel and with it, the possibility to investigate the surface and subsurface of other celestial bodies. Given the very high number of celestial bodies in our universe, it is essential to focus the search for extraterrestrial life to celestial bodies that are physico-chemical considered as habitable. Until now, telescopes like Keppler and TESS discovered already 61 exoplanets that are located in a habitable zone (Arecibo, 2022). In this context, a habitable zone means the presence of sufficient atmospheric pressure and temperature at which liquid water can exist. However, whether a planet or a celestial object is or ever was habitable depends not only on the presence of liquid water, but also on its current or historical physico-chemical conditions.

The ongoing discovery on Earth of numerous extremophiles, microorganisms capable of surviving extreme harsh environmental conditions, make some scientist believe that there is no place on Earth where live cannot exist. However, no one will deny that nothing can survive inside a solid granite block or inside red hot lava spewed out by an active volcano. Demonstrating that even surface or subsurface waterbodies can be as lifeless as the inside of a rock is not easy and demands multiple lines of evidence before it can be stated in a scientific acceptable and convincing way (McKay, 2014).

It is always tempting to suggest the presence of life, when some biological related molecules, signals or indicators are detected. Yet, a call for very cautionary interpretation of presumed positive biological signals where one or more physico-chemical parameters clearly exceeds the current known limits of habitability (Grant, 2004; Hallsworth et al., 2007; McKay, 2014; Stevenson et al., 2015). One must always take into account that earth’s atmosphere is far from sterile and explore all the numerous ways of unwanted external contaminations with living or dead microbes, biological debris, biological contaminations linked to the used methodology or materials that are prone to render false positive results (Kellogg and Griffin, 2006; Lee et al., 2012; Barberan et al., 2014; Maki et al., 2015; Amato et al., 2017; Weil et al., 2017; Behzad et al., 2018). Besides, certain physico-chemical conditions, like extreme high salinity, can be lethal for some living entities but can on the other hand enhance the fossilization process or preserve biology related structures and macromolecules, like proteins, nucleic acids or phospholipids for extended periods of time (Schreder-Gomes et al., 2022).

The halo-volcanic Dallol complex, located in the north east of the Danakil depression (Ethiopia), harbors dozens of the most extreme, hypersaline water bodies that exist on Earth (Gebresilassie et al., 2011; Perez and Chebude, 2017; Cavalazzi et al., 2019). Hypersaline waterbodies can only be present in regions having a salty geology. Evaporation is the main process for the formation of surface salt layers. But, the deep salty geology of the halo-volcanic Dallol complex is the result of ancient geological salt forming serpentinization reactions and hydrothermal activity as a consequence of the enormous subsurface heat generated by the locally ongoing continental rifting process and the close proximity of a magma reservoir (Schofield et al., 2014; Hovland et al., 2015; Warren, 2016; Pinto et al., 2017; Scribano et al., 2017; Hovland et al., 2018a,b; Cheng et al., 2019; Debure et al., 2019; Voisin et al., 2020). At the surface of the Danakil depression, thousands of years of intense solar radiation evaporated most of the remaining free surface water. The current subsurface volcanic activity still causes ascending aquifer flows of superheated water and on some locations even molten salts. The origin of the water of the ascending aquifers can be pure meteoric water, saline Red sea water or a mixture of both. Depending on the governing local physico-chemical conditions, encountered by the water flows during their rising flow path to the surface, the water can gain, salting in, or lose, salting out, substantial amounts of mineral substances of the passing salty host rock layers. When these hot saline mixtures reach the surface they give rise to a diversity of hydrothermal springs that feed basins of small waterbodies or in some cases even large lakes.

During our Danakil field expedition, in which we attempted to gain insight between extreme geology and the presence of life, 10 of these hyper saline waterbodies were extensively studied by *in situ* and *ex situ* geo-physico-chemical measurement methods, laboratory and microbiological analyses of collected samples, liquids as well as minerals and sediments collected from the shores.

## 2. Materials and methods

Different analytical methods were used to elucidate the environmental physico-chemical conditions that either allow or prevent microbiological life from being present. In view of accurate and representative geochemistry and geomicrobiology, special attention was given for the optimal preservation of liquid as well as solid samples. We performed *in situ* and *ex situ* physico-chemical water analyses. Mineralogy of the solid shore samples was analyzed by lithogeochemistry, whole rock analyses, and XRD and SEM–EDX investigations. The presence or absence, viability and activity of microbes, of liquid as well as solid samples, was analyzed by extra- and intracellular ATP measurements, DNA extractions of in the field obtained filtrates, residues of filtered liquid samples and dissolved solid mineral samples in our laboratories followed by 16S rDNA microbial community fingerprinting. To check microbial viability, cultivability and possible isolation, different microbial culturing techniques were attempted. Besides, SEM–EDX sample preparations were also optimized to allow the visualization of possibly present biological structures.

### 2.1. Geographic localization and *in situ* physico-chemical measurements

**Figure 1:**
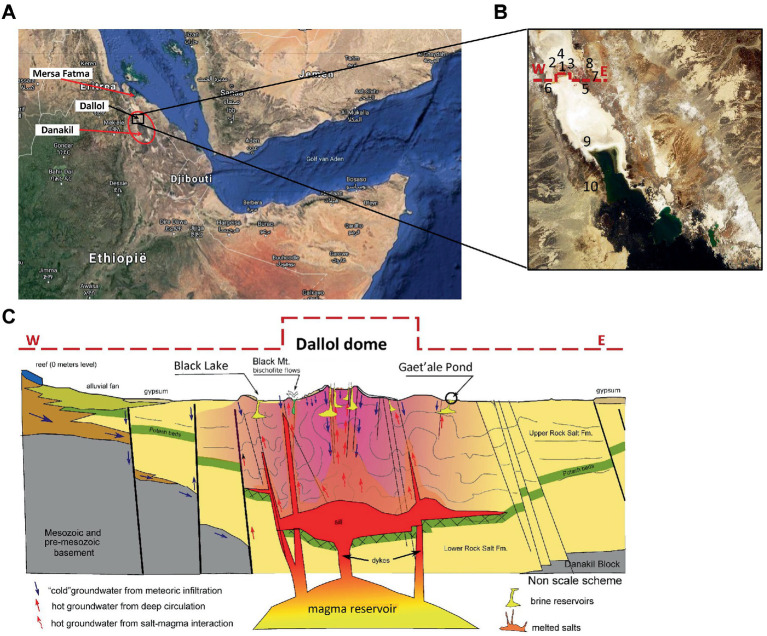
Overview of locations of the 10 sampled waterbodies of the halo-volcanic Dallol complex. Horn of Africa **(A)**, Zoom in on the locations of the 10 waterbodies, marked with numbers **(B)** and Transverse geological cross section, west to east, of the halo-volcanic Dalloll complex reproduced with permission and adapted from Lopez-Garcia et al. (2020) **(C)**.

All liquid and solid samples were collected during our Danakil expedition carried out in January 2018. An excel list of all samples that were taken can be consulted in the Supplementary material. Localization of samples and *in situ* physico-chemical measurements were performed with a handheld multi-parameter measuring device, Hanna 9828, equipped with a build in GPS locator. A robust measuring probe, housing a series of rugged chemical field electrodes, was connected by means of a 5 m long watertight cable to the multi measuring device. The Hanna 9828 is capable of performing seven parallel physico-chemical measurements: Dissolved Oxygen (DO), pH, Oxidation Reduction Potential (ORP), Ionic Electrical Conductivity (IEC), Temperature (T), atmospheric pressure, and solution density. Functionality of the Hanna 9828 multi-meter was verified and thoroughly tested by measuring reference solutions, after multi point calibrations of the DO-, pH-, and IEC-measuring probes. The ORP, T, and pressure measuring probes were extensively tested in our home laboratories before the start of the expedition. Even the GPS function was tested at numerous places by comparing the displayed latitude and longitude coordinates with a multifunctional mobile Garmin Nuvi 500 GPS. During the field expedition, the pH and IEC were occasionally verified with NIST traceable reference solutions that were individually packed in small hermitically heat sealed Al-PE poaches (Hanna reference solutions). Figures 1A–C, shows a global map view, a zoom in view, showing the numbered locations, as they also appear in Tables 1–3, of the 10 hyper saline waterbodies that were sampled and a transverse geological cross section, adapted and reproduced with permissions, of the halo-volcanic Dallol complex of the sampling site (Lopez-Garcia et al., 2020).

**Table 1:** Overview of the GPS locations, general *in situ* and laboratorium physico-chemical determinations of brine densities, TDS, and water activity of the 10 waterbodies of the halo-volcanic Dallol complex.

Parameter	Central green pool (1)	Left green pool (2)	Right yellow pool (3)	White chimney environment (4)	Gaet’ale Pond (5)	Black lake (6)	Yellow mini pond (7)	Red micro pond (8)	Asale karst hole (9)	Karum Lake (10)
GPS Latitude (North)	14°14′10.8″	14°14′10.6″	14°14′10.8″	14°14′11.5″	14°12′48.1″	14°13′18.4″	14°13′40.4″	14°13′41.7″	14°06′57.6″	14°02′58.0″
GPS Longitude (East)	40°18′00.5″	40°18′00.2″	40°18′00.8″	40°17′59.5″	40°19′17.1″	40°17′10.6″	40°19′08.1″	40°19′06.3″	40°20′53.2″	40°22′32.2″
*in situ measurements*										
Temperature [°C]	46.1	34.0	35.5	>100	41.2	35.7	32.0	35.1	35.5	31.5
*in situ* pH	−0.5	−0.5	−0.4	−1.1	6.2	2.4	4.7	3.1	5.6	5.4
Conductivity [mS.cm^−1^ at 25°C]	> max.	> max.	> max.	> max.	> max.	> max.	> max.	> max.	> max.	> max.
E_H_ [mV vs. SHE]	556	569	595	466	336	396	296	434	382	392
*Labo measurements*										
pH_1/50 diluted_ [−]	0.7	0.8	0.9	0.7	3.4	2.8	3.7	3.5	4.4	4.5
Conductivity [mS.cm^−1^]	422	418	404	424	598	521	395	494	373	375
E_H_ [mV vs. SHE]	620	619	630	627	460	478	396	426	407	416
Density [Mg.m^−3^]	1.225	1.223	1.222	1.223	1.454	1.351	1.246	1.342	1.220	1.218
TDS measured [g.l^−1^]	314	319	320	318	682	434	373	504	352	350
Water activity A_w_ [−]	0.725	0.728	0.731	0.729	0.234	0.324	0.684	0.463	0.734	0.735

**Table 2:** Summary of the measured major cat– and anions concentrations of the brine samples that were taken of the 10 different waterbodies of the halo-volcanic Dallol complex.

Parameter	Central green pool (1)	Left green pool (2)	Right yellow pool (3)	White chimney environment (4)	Gaet’ale Pond (5)	Black lake (6)	Yellow mini pond (7)	Red micro pond (8)	Asale karst hole (9)	Karum Lake (10)
*Major cations, ICP-MS [mM]*										
Na^+^	3,893	3,846	4,153	3,746	58	88	2,091	245	3,519	4,149
Mg^2+^	156	157	148	128	1,925	4,705	1,106	2,016	124	115
Ca^2+^	85	84	79	80	4,048	207	1,267	2,685	434	424
K^+^	195	197	168	157	40	46	260	322	90	88
Fe^2+/3+^	273	280	226	221	1	27	10	4	0.3	0.3
Sr^2+^	1	1	1	1	34	3	11	30	4	4
[B(OH)_3_]^0^	17	15	12	15	8	4	3	6	1	1
*Major anions, IC [mM]*										
Cl^−^	5,834	5,665	5,857	5,602	12,168	10,077	6,767	10,704	5,211	5,887
F^−^	18	16	14	17	<	<	4	11	<	<
Br^−^	3	3	3	3	95	116	30	67	10	9
SO_4_^2−^	58	54	42	61	<	<	2	<	3	4
PO_4_^3−^	<	<	<	<	<	<	<	<	<	<
NO_3_^−^	<	<	<	<	<	<	<	<	<	<
NO_2_^−^	<	<	<	<	<	<	<	<	<	<
HCO_3_^−^	<	<	<	<	4	<	<	<	4	5
CO_3_^2−^	<	<	<	<	<	<	<	<	<	<
Charge balance [mEq]	−836	−690	−717	−966	49	−171	276	−739	−492	−579
Chao-kosmotropicity [kJ.kg^−1^]	−19.7	−19.0	−24.8	−21.3	476.1	272.6	150.9	350.2	7.0	−1.3
TDS analyzed [g.l^−1^]	336	326	337	333	651	494	380	564	294	332

**Table 3:** Overview of the mineralogy of the samples collected of the shores of the 10 waterbodies of the 10 different waterbodies of the halo-volcanic Dallol complex.

Parameter	Central green pool (1)	Left green pool (2)	Right yellow pool (3)	White chimney environment (4)	Gaet’ale Pond (5)	Black lake (6)	Yellow mini pond (7)	Red micro pond (8)	Asale karst hole (9)	Karum Lake (10)
*XRD-mineral analysis*	NaCl, KCl, CaSO_4_.12H_2_O, and Ca(Mn,Mg)(CO_3_)_2_	NaCl, KCl, CaSO_4_.12H_2_O, and Ca(Mn,Mg)(CO_3_)_2_	NaCl, KCl, CaSO_4_.12H_2_O, and Ca(Mn, Mg)(CO_3_)_2_	NaCl, KCl, CaSO_4_.12H_2_O, Ca(Mn,Mg)(CO_3_)_2_, and CaSO_4_	NaCl, KCl, CaMg_2_Cl_6_.12H_2_O, and MgCl_2_	MgCl_2_.6H_2_O, and KMgCl_3_.6H_2_O	NaCl, CaMg_2_Cl_6_.12H_2_O	KCl, KMgCl_3_.6H_2_O	NaCl, KCl	NaCl, KCl
	Halite, sylvite, gypsum, and kutnohorite	Halite, sylvite, gypsum, and kutnohorite	Halite, sylvite, gypsum, and kutnohorite	Halite, sylvite, gypsum, kutnohorite, and anhydrite	Halite, sylvite, tachyhydrite, and chloromagnesite	Bischofite, carnalite	Halite, tachyhydrite like	Sylvite, carnalite	Halite, sylvite	Halite, sylvite

### 2.2. Liquid sample handling

All liquid samples were collected in pre-cleaned and -sterilized 50 mL glass septum bottles, mounted and fixed on the top of a 6 m long telescopic carbon rod, submerged into the extreme brine solutions. Supplementary Figure S1 illustrates the collection of liquid samples. Filled septum bottles were removed from the brine and were immediately hermitically closed by a sterile, 13 mm tick butyl rubber septum. The septum was manually forced into the fill opening of sample bottle, thereby always avoiding the intake of any atmospheric gas. The result was a closed hermetic sealed septum bottle in which no gaseous headspace was present. All septum bottles closed with rubber septa were on the spot additionally sealed by forcibly crimping an aluminum closure ring on top of the glass bottle. This *in situ* liquid sampling procedure guaranteed that the liquid samples enclosed inside the vial remained geochemical– and microbial undisturbed throughout the whole period of on-site handling, custom clearances, long term airplane transportation, and controlled storage in our analytical laboratory.

### 2.3. Solid samples handling

All sediment, solid and mineral samples were taken by hand picking of samples, wearing umonium sterilized nitrile chirurgical gloves. Whenever needed, a geological hammer was used to break and isolate solid samples from the main geological host formation. After isolation, the solid samples were immediately stored into aluminum-poly ethylene coated sample bags. Upon each daily return to the base camp, all Al-PE packed solid samples bags were made vacuum and subsequently heat-sealed to obtain a fully isolated and gas tight packaging of solid samples as can be seen in Supplementary Figure S2. This sample treatment effectively prevented any further exposure of the solid samples to the atmosphere thereby limiting unwanted mineral transitions, like oxidation, atmospheric gas interactions, or water uptake. The latter can lead to detrimental and unwanted mineral transformations. For instance, due to its deliquescence, i.e., excessive moisture uptake whereby the mineral dissolves in its own crystal water, the unstable mineral tachyhydrite (CaMg_2_Cl_6_.12H_2_O) is known to transform into a Ca-rich mother liquor and the mineral bischofite (MgCl_2_.6H_2_O) when simply exposed to standard humid atmospheric conditions (Cheng et al., 2019). Our *in situ* isolation system of solid samples, by storing the solid samples into vacuum hermetic heatsealed Al-PE sample bags, assured an excellent mineral integrity and unaltered geochemistry during overseas transportation and long storage periods.

### 2.4. Brine solution density measurements

To determine the density of the liquid brine samples, a direct gravimetric analysis was applied. This density analysis is based on the accurate weight of a known volume of brine sample at a given temperature. Densities were determined at room temperature. An electrical powered and calibrated positive displacement pipet (Eppendorf AG, Germany) was used to transfer a volume of 1 cm^3^ into a small glass petri dish. The corresponding weight of the brine sample was measured with an analytical scale, Sartorius BCE224-1S Entris® II (Sartorius, Germany). This procedure was performed on brine samples taken at various locations in each of the 10 investigated Dallol waterbodies. Every density analysis of each individual brine sample was performed in triplicate.

### 2.5. Determination of water activity of brine solutions

The measurement of the water activity (A_w_) was performed with a resistive electrolytic hygrometer NovasinaTouch™ (Novasina AG, Suisse). The sensor of this water activity meter is a solidified gel electrolyte that is held between two very small glass rods by capillary force. The electrical resistance of the gel electrolyte is measured. This electrical resistance is very sensitive to small changes of the relative air humidity inside a temperature controlled measuring chamber. The chronological evolution of relative air humidity was monitored over time until a steady signal was obtained. The final signal corresponds with the vapor–liquid equilibrium of the sample inside the measuring chamber. The NovasinaTouch™ apparatus converts the signal directly into the A_w_ value of the sample.

### 2.6. Gravimetric analysis of total dissolved salts

We also performed the standard indirect gravimetric analysis of the Total Dissolved Salts, TDS, of samples of the original brine solutions. This standard methodology to determine TDS of liquid samples consists of evaporating a sample to dryness by heating the sample in a ventilated oven to a temperature of about 110°C. However, due to the extreme high salt content of the Dallol brine samples it was impossible to obtain complete dryness. Most samples remained tick jelly like fluids, even after attempting to dry the samples for extended periods of time, at 110°C. We were forced to adapt the standard drying protocol by increasing the drying temperature to more than 240°C to ultimately obtain dry residues. Supplementary Figure S3 shows the outcome of our samples after applying the adapted drying protocol at 240°C. Supplementary Figure S3 also shows another major interference when determining TDS by a drying protocol. Reduced chemical species get oxidized introducing a clear oxygen bias on the weights recorded of dried samples. As an extra precaution, our dried samples were cooled down in a desiccator and immediately weighed to obtain stable and representative dry weights of the residues and consequently a more accurate result of the calculation of the measured TDS values.

### 2.7. Chemical analysis of brine solutions

All of the chemical analyses of the liquid samples were outsourced to Activation Laboratories Ltd. (Ancaster, Ontario, Canada). Activation Laboratories Ltd. has achieved the ultimate accreditation to international standards, with either ISO 17025 for specific registered tests or certification to ISO 9001:2008. ISO 17025 evaluates the quality system and specific analytical methodologies through proficiency testing and routine audits of the laboratory. The brine samples of the Dallol ponds were diluted with MilliQ water according to the guidelines of Activation Laboratories Ltd. These guidelines demand that brine samples had to be diluted until the salinity was lower than 0.05%. Such dilution is needed to allow accurate chemical analyses and to avoid the need for substantial measurement corrections that would be needed to be calculated if undiluted original brine samples would have been analyzed. It also allows to ignore the impact of substantial variations of brine densities on the chemical results. It is noteworthy to mention that the correct conversion of fluid densities in the chemical calculations, especially for high concentrated brine samples, are of the utmost importance when determining the analytical concentrations of the original samples.

To avoid chemical, biochemical, and microbiological biases during transport and analyses, all samples were treated according to the sample treatment recommendations of Activation Laboratories Ltd. Additionally. Diluted samples were sterilized with a sterilization method that did not generate an extra bias for the specific applied analytical technique. Samples designated for Inductive Coupled Plasma—Optical Emission Spectrometry/Mass Spectrometry (ICP-OES/MS) were gravimetrically diluted with MilliQ water on a mass-to-mass ratio. The dilution range was predefined between 50- and 100-fold. After dilution, the samples were chemically stabilized for accurate analysis by acidification to a pH of about 2.0 with specific ICP-MS grade HNO_3_. Heat sterilization, autoclaving for 20 min at 120°C, was performed in hermitic closed glass septum bottles. The use of hermitic closed glass septum vials guarantees the preservation of the chemistry of the samples. The bottles were only opened by Actlabs analytical technicians immediately before the actual analysis.

Samples designated for Ion Chromatography and alkalinity were gravimetrically diluted with MilliQ water on a mass-to-mass ratio. Technical triplicates were made with a predefined dilution range between 50- and 100-fold. To avoid possible analytical biases and to preserve the chemistry diluted IC samples were heat sterilized by autoclaving (20 min at 120°C) in hermetic closed glass septum vials. Because heat sterilization can generate biases for alkalinity analyses, diluted alkalinity samples were only filter sterilized by membranes with pore sizes of less than 0.22 μm. The filtered sample solutions were transported to Activation Laboratories Ltd. in traditional sterile blue screw capped 50 mL Falcon tubes.

### 2.8. Chaotropicity determination

Chaotropicity was calculated based on the methodology and data provided by Cray et al. (2013, 2015). As chloride was by far the most dominant and almost exclusive anion present in all the Dallol samples (cfr. Table 2), all the salts of the present cations were considered to be chloride salts with a corresponding molar salt concentration equal to the sum of all cation concentrations, taking into account the correct stoichiometry coefficients.

### 2.9. Mineralogy of shore samples, lithogeochemistry, and whole rock analyses

The main techniques used to study the mineralogy of all the solid shore samples were: scanning electron microscopy (SEM) associated with Energy Dispersive X-ray analysis (EDX; ProX table top SEM–EDX, Phenom, Netherlands) and with X-Ray Diffraction (XRD) technique. Different subsamples of collected solid samples of the shores of Gaet’ale Pond, Black Lake and Red micro Pond were dried overnight in a ventilated furnace at a temperature of 240°C and afterward, individually grinded with a pestle and a mortar to obtain the reduced grainsizes that are necessary for SEM–EDX and XRD analyses.

Additional lithographic and whole rock analysis data of the solid subsamples of Geat’ale Pond and Black Lake were obtained by lithogeochemical analyses. These specific geological analyses were all outsourced to Activation Laboratories Ltd. that has achieved the CAN-P-1579 mineral analysis accreditation. Activation Laboratories Ltd. are one of the very few commercial laboratories that has achieved this important analytical distinction. All mineral samples were dried overnight in a well ventilated furnace at a temperature of 240°C. After drying, each mineral sample was individually crushed in a laboratory ball mill to render grains of which 95% has a size that is lower than 75 μm. The following lithogeochemical/whole rock analyses were performed: Lithium metaborate/tetraborate fusion ICP of whole rock combined with trace element ICP/MS analysis, InfraRed (IR) Carbon and Sulfur analysis, Neutron Activation Analysis (INAA) to determine Chlorine content and Ion Specific Electrode analysis of the fluoride content.

### 2.10. Microbiological analyses

Microbial presence or absence was determined by measuring the concentration of Adenosine-5’-TriPhosphate, ATP. We used the highly sensitive total and intracellular BioThema ATP kits HS (Isogen Life Science B.V., Netherlands). These kits apply the standard addition method to exclude any bias due to the impact of the sample matrix (e.g., coloration, organic solutes, high salinities, …). To measure the intracellular ATP concentration, i.e., a true measure of present and intact living cells, 50 μL sample was incubated with an equal amount of ATP eliminating reagent, thus neutralizing the potential presence of extracellular ATP, originating from contamination or from dead cell debris. Next, 50 μL of a cell lysis solution was added, which acts in a double way. It inactivates the ATP eliminating reagent and lyse viable cells. The latter causes a release of intracellular ATP. Afterward, the total volume was mixed with 400 μL of ATP reagent HS (containing D-luciferin). The immediate light intensity was measured with a Lumitester C-100 (Kikkoman). Then, 10 μL of 100 nmol.L^−1^ ATP standard was added and the immediate light intensity was measured again. Both measurement allow the accurate calculation of the concentration of intracellular ATP. The first step, the ATP elimination step, is omitted to determine the total ATP concentration of a sample. Strong interfering sample matrices were diluted to obtain meaningful measurement results.

Scanning electron microscopy–Energy Dispersive X-ray investigations of solid minerals, field filtrates and debris on filtered brine samples were pretreated to allow SEM analysis of biological materials. First, the samples were dehydrated using an ascending series of ethanol concentrations. Second, the samples were dried by submersing in a Hexamethyldislizane solution followed by air drying. Finally, the samples were sputter-coated with a gold layer of 5 nm using a LUXOR^Au^ goldcoater (Luxor Tech, Belgium). This pretreatment ensures the preservation and thus the visualization of microbes or any biological structure that might be present in a sample during the SEM–EDX analyses.

DNA was extracted, with our in house developed DNA extraction method, from microbes retained and concentrated on filters after 0.22 μm filtration of brine samples of all 10 hypersaline Dallol waterbodies and from dissolved solid salt samples taken from most of the shores (Wouters et al., 2013). Extreme viscous brines were preliminary diluted to facilitate the filtration process. To control the DNA quality, PCR amplifications of the 16S rDNA with the universal 8F and 1492R primers were executed. All samples that rendered a positive 16S rDNA amplicon were analyzed by the company BaseClear (Netherlands) for a 16S rDNA microbial community sequencing. The FASQ-datafiles containing the DNA sequences were treated with our new in house developed integrated bioinformatics pipeline, OCToPUS (Mysara et al., 2016b). This pipeline includes bioinformatics tools like: IPED for specifically denoising raw data of MiSeq (Mysara et al., 2016a), and CATCh for eliminating chimera sequences (Mysara et al., 2015). After passing the bioinformatics pipeline, we performed a literature survey of the biological background of the remaining consensus OTU reads and checked whether the most resembling species could be potentially and logically linked to the physico-chemical environment in which the it was discovered.

In parallel to the above techniques, attempts to cultivate microbes were carried out using six different halophile- and acidophile culture media (composition is given in Supplementary material): An own designed halophilic Dallol medium, called Simulated Asale medium (SAM), the reference halophilic *Salinibacter ruber* (DSMZ medium 936), and *Halobacterium* (DSMZ medium 97) media, an own adapted version of an acidophilic promoting *Sulfolobulus* medium (DSMZ medium 88), Seghal&Gibbons medium (DSMZ medium 1,336), typical medium used to isolate and enrich halophiles and, a own adapted version of the Postgate medium or DSMZ medium 63 to promote the growth of sulfate reducing microorganism (A63 medium). Culture attempts with the latter medium and medium 63 were performed in a glove box, under strict anaerobic conditions, i.e., 99% argon and 1% hydrogen. Potential growth was monitored by OD_600_ and by ATP measurements of all liquid cultures and by colony formation observation for culture attempts performed on agar plates. Inoculation of the media were done by pipetting, for liquid cultures, or spreading aliquots, for cultivations on solid agar, of liquid brine samples of all hypersaline Dallol waterbodies.

## 3. Results and discussion

### 3.1. Geography

Geographically, the 10 investigated hypersaline waterbodies of the halo-volcanic Dallol complex could be clustered in three clearly separated zones.

**Figure 2:**
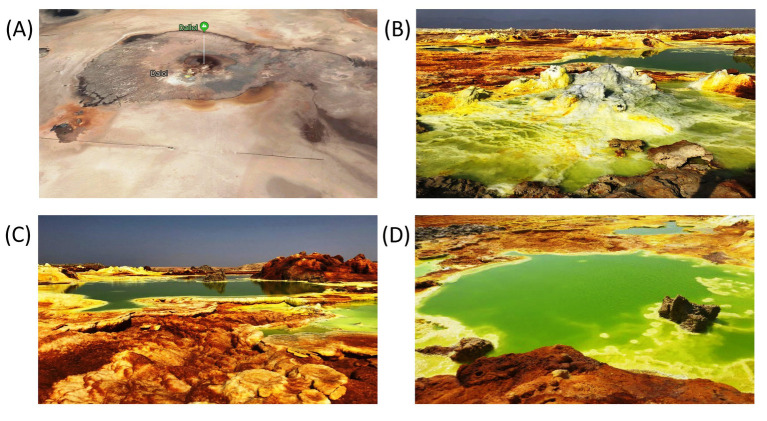
Typical waterbodies of the outcrop zone. Google earth satellite view of the Dallol outcrop zone **(A)**. Typical fumarole with waterbody **(B)**, Central green pool **(C)**, and Right yellow pool **(D)**, all located inside the krater of the halo-volcanic Dallol dome.

A first zone, the Dallol outcrop zone, harbored intense green or yellowish green colored waterbodies, either as stagnant brine pools or vividly fueled by water and steam spewing geysers (outflow temperatures were measured of up to 110°C). Figure 2 shows the waterbodies we investigated that were located in the outcrop zone. The water, coming out by these hot springs, contained huge amounts of reduced sulfur and iron species (cfr. Table 2). Both elements oxidizing very fast during the downward flows of the brines into the different basins. The top of most halite geyser mounds was white. An indication that the oxidation of sulfur or iron did not occur at the top of the mounds. Closer to the bottom of the halite mound, the color gradually changed to bright yellow, a color change most probably caused by the primary oxidation of sulfur species. Further down, at the lower base of the geysers, the color gradually changed to brown even evolving to intense dark brown. Most likely, a clear indication of the oxidation of ferrous compounds into ferric iron species. A recent study of Belilla et al. (2022) confirms our hypothesis on potential iron oxidation along the wall of halite geysers. Iron oxidation is exothermic thereby causing an additional increase of the temperature of the brine of the waterbody.

**Figure 3:**
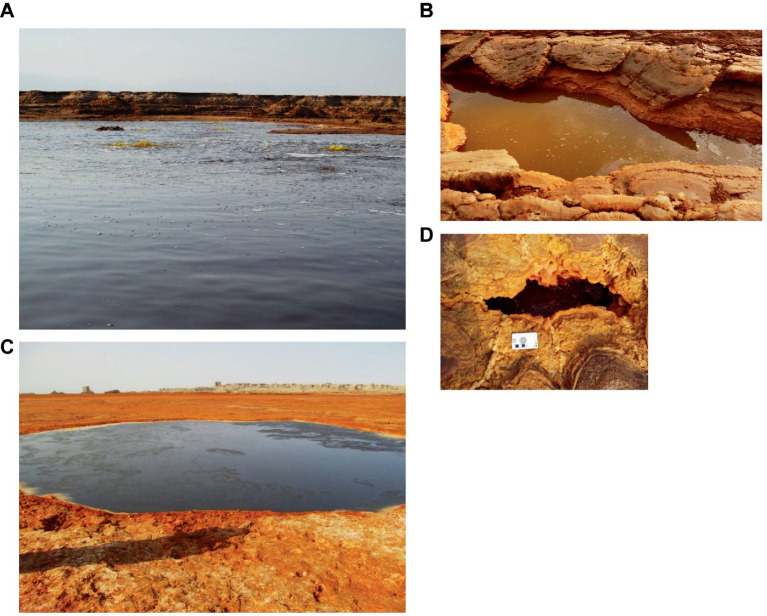
Waterbodies of the zone at the south south-east base of the Dallol dome, second zone. **(A)** Gaet’ale Pond. **(B)** Yellow mini pond, **(C)** Black Lake, and **(D)** Red micro pond.

A second zone, the zone at the south-southeast base of the halo-volcanic Dallol complex, harbored larger and apparently much deeper hypersaline waterbodies. Figure 3 shows pictures of the four waterbodies we investigated of the south-southeast base zone of the halo-volcanic Dallol complex. The two most dominating waterbodies of this zone were the Gaet’ale Pond, also known as the Yellow Lake, and the Black lake. At the moment of sampling, the source that fueled the Gaet’ale Pond was inactive. Only a huge amount of volcanic carbon dioxide gas underneath the Gaet’ale basin, vigorously sparged the brine solution, forcing it to physico-chemically equilibrate with this acidic gas. Obviously, this will have an important impact on the pH of the brine and consequently promotes the acid mineral dissolution and precipitation processes (Darrah et al., 2013; DePaolo and Cole, 2013; Babel and Schreiber, 2014; Zhu et al., 2019). The brine of the Black Lake was mildly agitated by either a thermal generated advection flow or an ascending brine flow from underneath its basin. At the surface of the Black Lake, a constant slow surface movement coincided with the formation and disappearance of a thin white salt crust layer. Perhaps this tempered brine agitation was a remnant of a subsurface active bischofite flow in contact with the brine solution, causing this dynamic mineralization-dissolution process (Warren, 2015).

**Figure 4:**
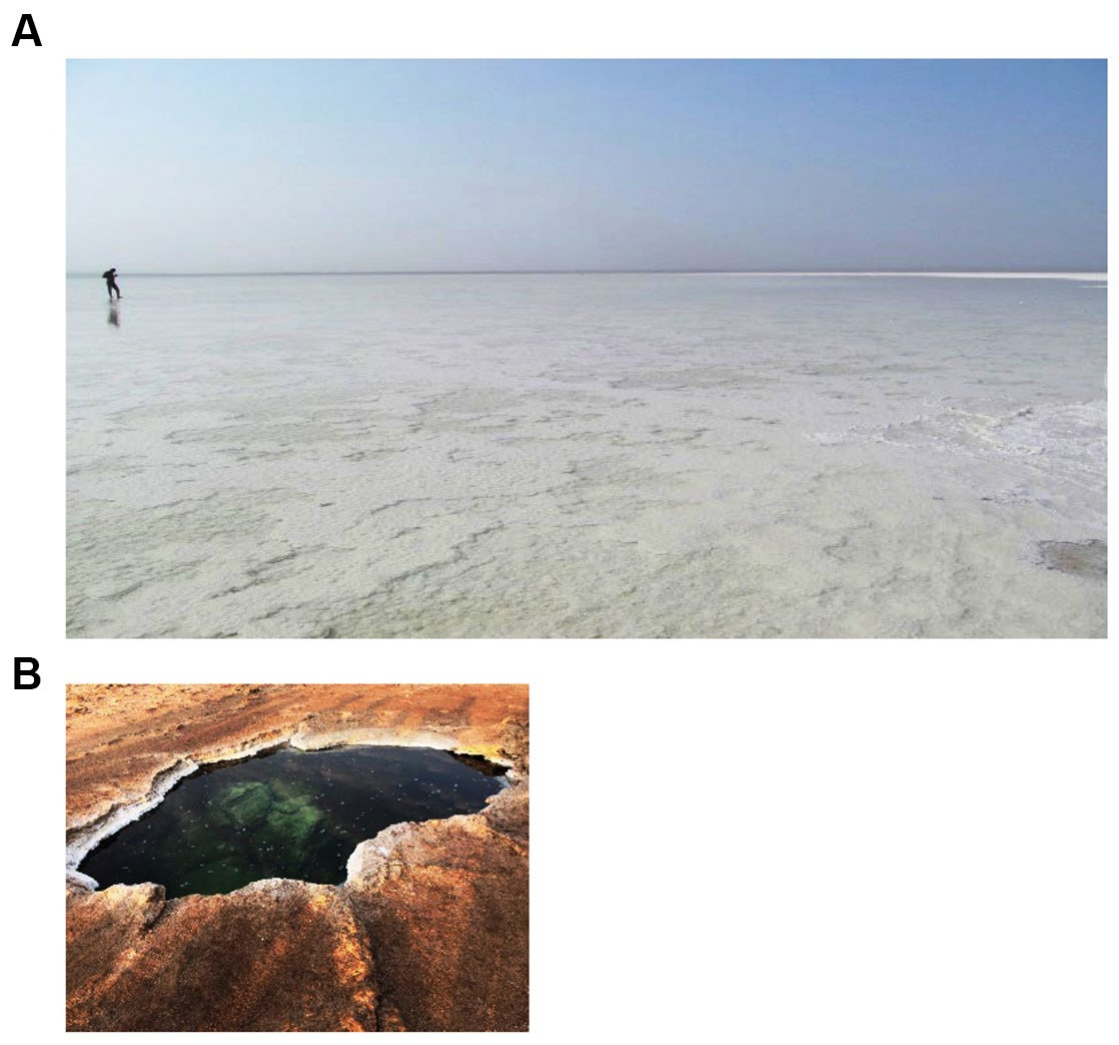
Waterbodies of the zone of the flat south plane, third zone. **(A)** Big Karum Lake and **(B)** Asale Karst hole.

A third zone, the zone of the flat south plane further away from the halo-volcanic Dallol complex, harbors two of the main waterbodies we sampled, the Asale karst hole and the very large but shallow Karum Lake. Figure 4 shows pictures of the two waterbodies of the flat south plane zone. Both lakes have tight links with human activity. The Asale karst hole is likely artificially created by forcibly breaking its natural covering salt curst thereby opening and contacting this subsurface small water reservoir to normal atmospheric conditions. Karum Lake is most likely the result of the accumulation of surface water in the lowest region of the halo-volcanic Dallol complex. Karum lake is currently being used for industrial salt mining (Cavalazzi et al., 2019). Both waterbodies are completely dominated by sodium chloride (cfr. Table 2).

### 3.2. Physico-chemical analyses

Although the Hanna 9828 analytical field device should be capable of accurately measuring *in situ* seven physico-chemical parameters, it rarely succeeded to display any meaningful measurement. Most brine solutions of the Dallol waterbodies were too extreme to be measurable with a Hanna 9828 field device. Laboratory physico-chemical measurements of diluted brine samples were needed to obtain a better insight of the status of the physico-chemical conditions of the Dallol water bodies and to better correlate the physico-chemistry with the geographical observed zonification (cfr. Table 1). At the time of sampling only one water system, the white chimney/fumarole environment, had a temperature higher than 100°C. The *in situ* measured pH values of all the waterbodies of the outcrop zone were negative, while the *in situ* pH values of the base zone and the flat salt plane were only mildly acidic. As expected for volcanic active zones, *in situ* redox values indicated strong oxidizing solutions. All instrumental laboratory measurements of the diluted brine samples confirmed the extreme nature of the original brine solutions.

### 3.3. Brine chemistries

**Figure 5:**
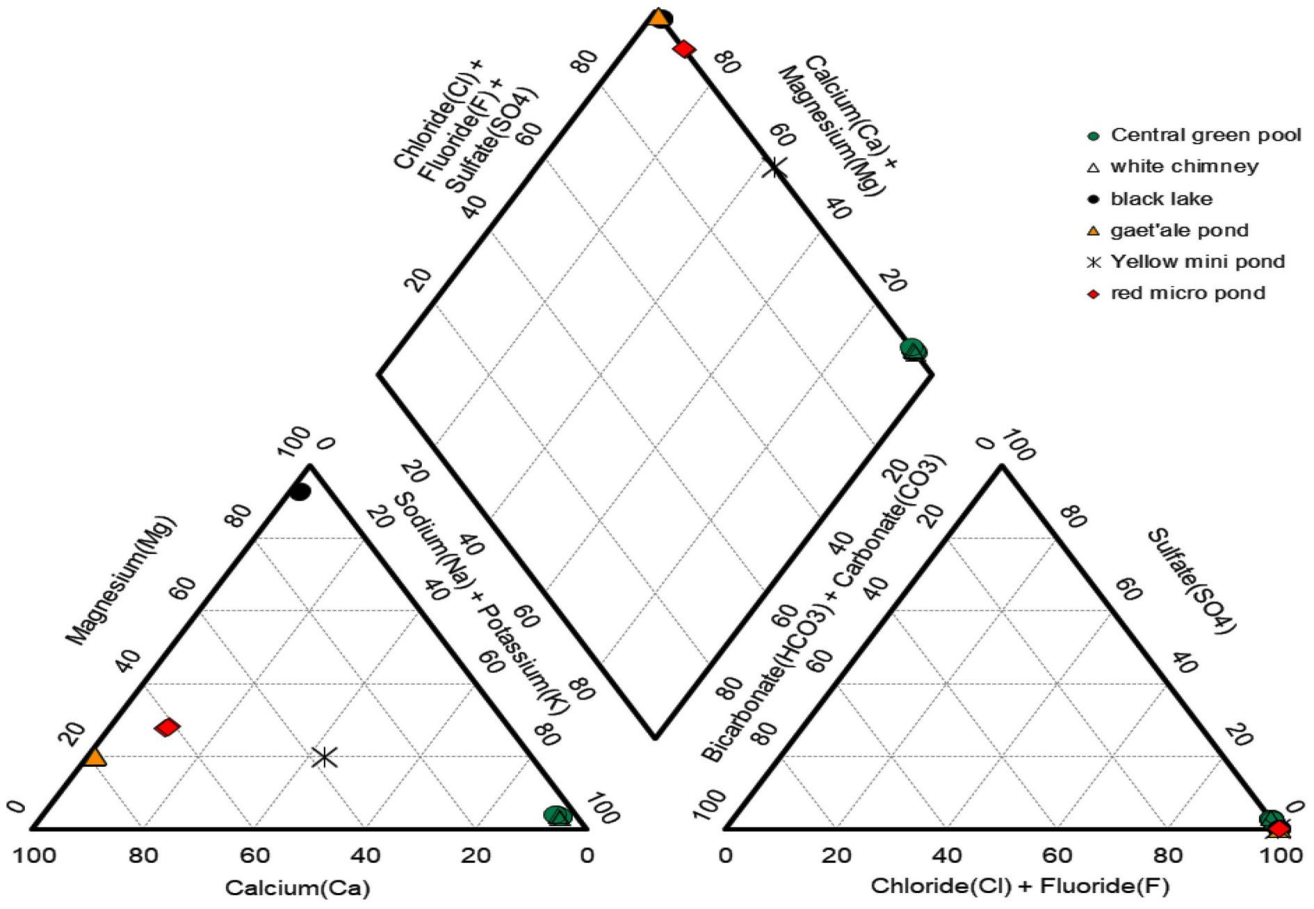
Piper diagram presenting the relative distribution of the major cat- and anions of six of the 10 waterbodies of the halo-volcanic Dallol complex.

The concentrations of the major cat- and anions are given in Table 2. Figure 5 visualizes the extreme brine chemistries in a chemical Piper diagram. The Piper diagram reveals clearly that chloride is the most dominant anion of all waterbodies. The major differences of the brine chemistries are related to the different cations, monovalent versus divalent. As a quality control factor, Table 2 also presents the ion charge balance of each brine. The calculated Chao-Kosmotropicity data and the analytical TDS values are presented in the last two rows. The major ion concentrations reveal three main types of solution chemistries, which coincide with the three observed geographic zones. The waterbodies of the outcrop zone are characterized with very hot and highly concentrated NaCl brines, rich in iron and sulfur. Oxidation processes in the outflowing superheated ferrous- and sulfidic rich source solutions give rise to some of the most acidic and colorful brines on our planet, as can be witnessed on Figure 2. The brines of the waterbodies of the south-southeast base of Dallol were clearly dominated by the divalent cations, Ca^2+^ and Mg^2+^. The extreme high salinity, especially of the Gaet’ale Pond, gave rise to very high solution densities and viscosities, thereby giving its brine an pronounced oily look and feel. It is noteworthy to mention that these Dallol base brines also contain substantial Sr^2+^ concentrations. Like the waterbodies of outcrop zone, the two waterbodies of the flat south plane are dominated by sodium chloride. Asale karst hole and Karum lake are different as they contain a higher Ca^2+^ concentration (±430 mM) and a 1,000-fold less iron concentration (±0.3 mM). Besides, the pH was only mildly acidic pH (±5.5). These differences make the Asale karst hole and the Karum lake, the least chemical aggressive waterbodies.

**Figure 6:**
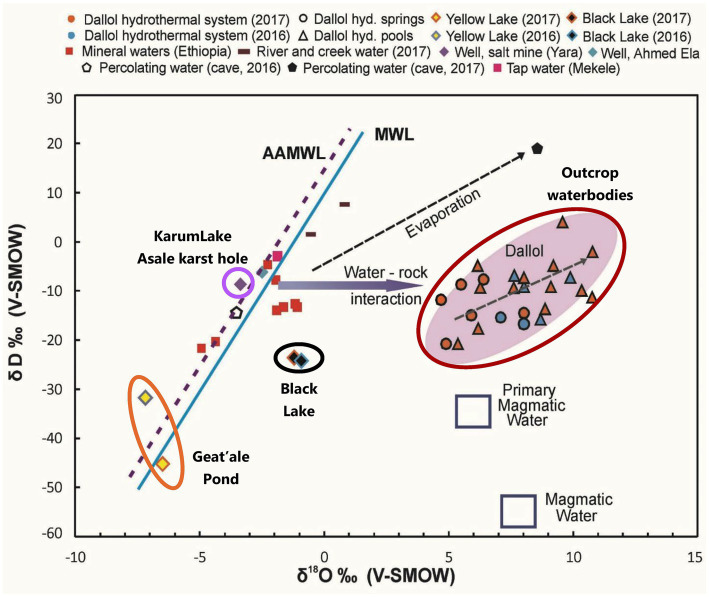
Stable isotopes analysis indicating the different and distinct origin of the waters of the halo-volcanic Dallol complex. With permission and adapted from Kotopoulou et al. (2019).

**Figure 7:**
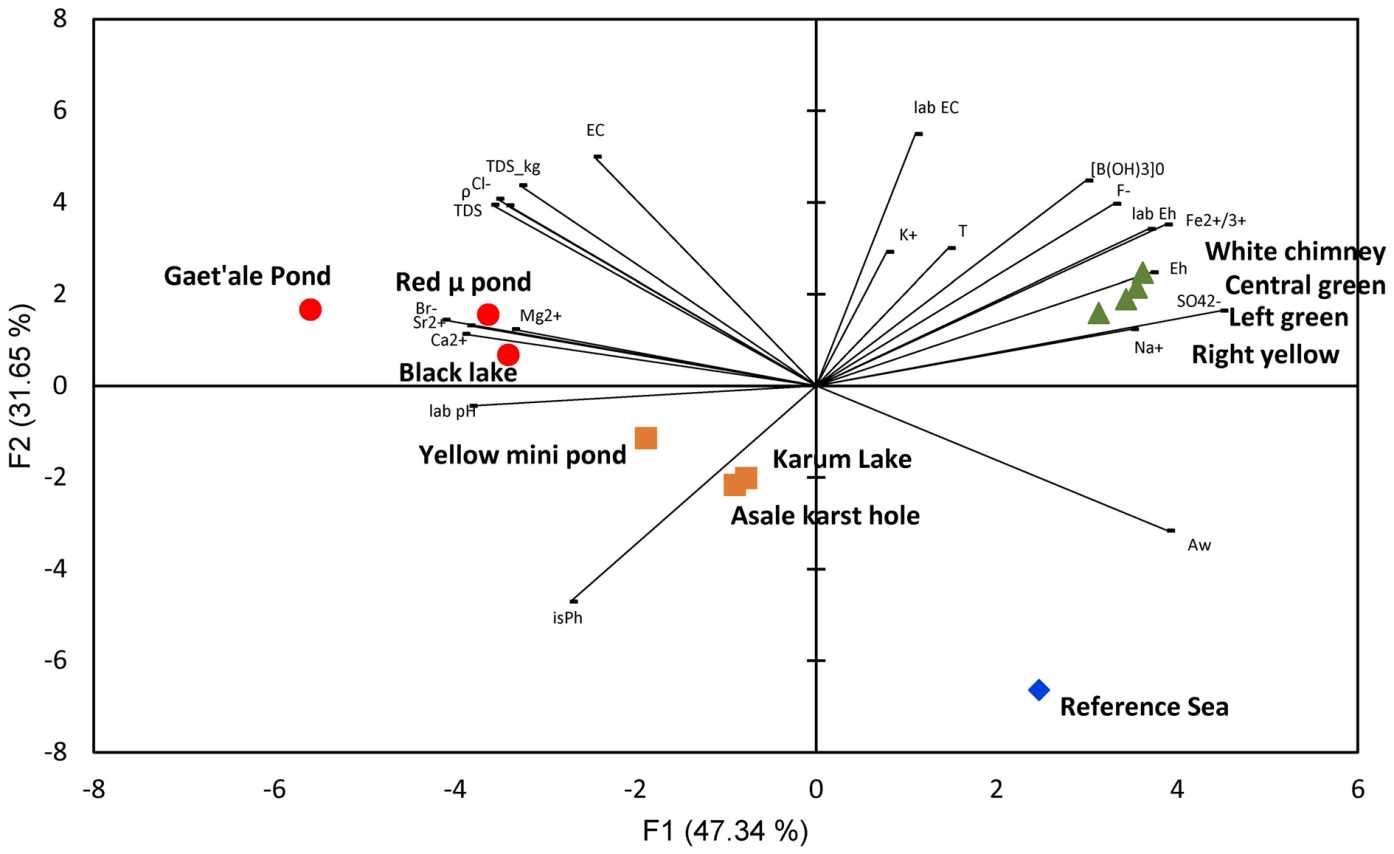
PCA analysis showing the clustering of the 10 waterbodies of the halo-volcanic Dallol complex into three groups based on 22 physico-chemical parameters.

Stable isotope data and principal component analysis (PCA) provided two additional strong indications that the brines of the Dallol waterbodies could indeed be clustered into three very different geochemical groups, spread over three different geographical zones. Stable isotope data obtained by Kotopoulou and collaborators are visualized in Figure 6. Their data clearly shows the different origins of the source waters of the waterbodies of the three separate geographic zones (Kotopoulou et al., 2019). Our PCA presented in Figure 7 indicates also that the Dallol waterbodies can indeed be divided into three different categories. The Yellow mini pond is the only waterbody that correlates somewhat less with the other waterbodies of the south-southeast base zone.

### 3.4. Mineralogical correlations

Our mineralogical determinations, presented in Table 3, shows that the minerals, found on the shores of the waterbodies in the three different zones, are in full agreement with the observed brine chemistries. Additional SEM pictures of the minerals of the waterbodies of the halo-volcanic Dallol complex are presented in Supplementary Figures S4–S6 of the Supplementary material. The outcrop zone was dominated by the minerals: halite (NaCl), sylvite (KCl), gypsum (CaSO_4_.2H_2_O), and kutnohorite [Ca(Mn/Mg)(CO_3_)_2_]. Around the extreme hot white chimney also some anhydrite (CaSO_4_) was observed. The waterbodies of the south-southeast base zone were surrounded by the minerals: halite, sylivite, tachyhydrite (CaMg_2_Cl_6_.12H_2_O), and chloromagnesite (MgCl_2_). Carnalite (KMgCl_3_.6H_2_O) was observed near the Red micro pond and the shores of the Black Lake. The shores of the latter, however, were mostly dominated by bischofite (MgCl_2_.6H_2_O). The flat south plane zone contained only two types of minerals, halite and sylvite. The observed mineralogy in the vicinity of the Dallol waterbodies was, without any exception, in good agreement with the observed governing chemical parameters of the different brines.

### 3.5. Microbiology of Dallol

From the above data, it is clear that the halo-volcanic Dallol complex is a geological area that can be subdivided into three very different geochemical zones. Based on the physico-chemical data and the current existing literature on the limits of life, we expected that the waterbodies of two zones, the outcrop and the south-southeast base zone, would be lifeless (Hallsworth et al., 2007; Tosca et al., 2008; McKay, 2014; Stevenson et al., 2015). Only the flat south plane shows a physico-chemistry, i.e., temperature, pH water activity, chao-kosmotropicity, salinity, and ionic activity that might allow inhabitation by certain halophilic extremophiles. To verify our hypothesis of uninhabitable and inhabitable zones, we carried out a broad spectrum of non-culture and culture microbial techniques. All our measured internal and total ATP concentrations of liquid as well as solid samples were either below the limit of detection or just above. However, it is noteworthy to mention that the extreme chemistries of the brine samples hindered the ATP measurements. A 200-fold dilution had to be made before meaningful ATP measurements could be performed. Such high dilutions also had an impact on the limit of detection of the ATP measurements. We calculated a limit of detection of 1.4 × 10^−14^ Mol ATP for the diluted brine samples which was 3 orders of magnitude higher than expected.

Multiple attempts of DNA extraction have been undertaken. We were able to extract PCR grade of DNA from 10 samples out of the total of 25 original *in situ* taken liquid samples. Four DNA samples from brines of the waterbodies of the outcrop zone, three DNA samples from brines of the south-southeast base zone, and three DNA samples from the flat south plane zone. We outsourced the 16S rDNA microbial diversity analyses to the company BaseClear BV (Netherlands). As the used primers in the standard methodology of BaseClear preferentially bind bacterial DNA, the detection of possible present archaea was hampered. Even the results of the second analysis, for which the company claimed to have used better archaea suited primers, was still largely dominated by bacterial 16S rDNA sequences. Our bioinformatics pipeline, OCToPUS, eliminated almost 80% of all the raw DNA reads based on its build in denoising- and chimera elimination algorithms. The remaining reads were clustered in 498 meaningful OTU’s of high data quality. Details of the results of our analyses can be consulted in Supplementary material. Our biological literature survey revealed that most of the detected OTUs were likely the result of DNA contaminations typically found in substances of molecular biological kits, present on standard laboratory materials or introduced as aerosols during execution of laboratory procedures (Sheik et al., 2018; Weyrich et al., 2019). Many other OTUs were also likely the result of contaminations. But contaminations of DNA’s that were introduced *in situ*, in the field, before sampling took place. This DNA was most probably introduced by occasionally passing eukaryotic animals like insects, bats or migrating birds, during activities of the local Afar population, as a consequence of the intensive tourist visits to the Dallol site, deposited by the annual floodings or simply as airborne microbial transmissions by clouds and dusts carried by winds and storms (Kellogg and Griffin, 2006; Barberan et al., 2014; Master, 2016; Amato et al., 2017; Gat et al., 2017; Weil et al., 2017; Behzad et al., 2018; Tignat-Perrier et al., 2019; Belilla et al., 2022). A full analysis of the results of the 16S rDNA analyses can be consulted in Supplementary material. Only a very limited amount of the detected OTUs, mostly belonging to archaea, could be designated as most likely indigenous species to the geosphere of the halo-volcanic Dallol complex. Table 4 lists the data and Supplementary Figure S7 shows a distribution of the OTU’s detected in the brines of the Asale karst hole and Karum lake of which a number are known as true halophylic extremophyles. As can be noted on Supplementary Figure S7 and Table 4, five OTU’s were detected simultaneously in both waterbodies of the South plane zone.

**Table 4:** Overview of the most dominant (> 1% occurance) and possible indigenous microbial OTU’s detected in the original brines of the Asale karst hole and the Karum lake.

OTU #	Group description	Asale karst hole (9)	Karum lake (10)
17	Salinibacter	47%	26%
31	Deltaproteobacteria_unclassified	15%	17%
48	Bacteroidetes_unclassified	4%	13%
38	Bacteria_unclassified	14%	0%
91	Bacteria_unclassified	2%	10%
102	Saccharothrix	0%	4%
125	Methylobacterium	0%	2%
126	Bacteria_unclassified	0%	2%
143	Salinibacter	0%	2%
69	Candidatus_Nanosalina_unclassified	2%	9%
61	Candidatus_Nanosalina_unclassified	5%	0%
89	Candidatus_Nanosalina_unclassified	5%	0%
127	Candidatus_Nanosalina_unclassified	0%	2%
191	Candidatus_Nanosalina_unclassified	0%	1%
Others		5%	13%

Five OTU’s occurred in both waterbodies.

Almost all of the numerous cultivation attempts in liquid and on solid agar, using the original brine solutions as inoculum, rendered negative results. We only obtained positive signs of life, i.e., positive ATP results in the cultivation attempts with aliquots of brines of Asale karst hole and Karum lake, the south-southeast base zone, using liquid SAM and 936 medium. All attempts to transfer positive liquid SAM and 936 culture onto solid SAM and 936 medium plates, to obtain isolated colonies, failed. Therefore, we performed DNA extractions followed by 16S rDNA analysis of the positive liquid media. Among the many most likely laboratory contaminants, like bacillus species, also DNA sequences of *Limimonas* and canditatus *Nanosalina* were detected in the medium of the liquid cultures.

**Figure 8:**
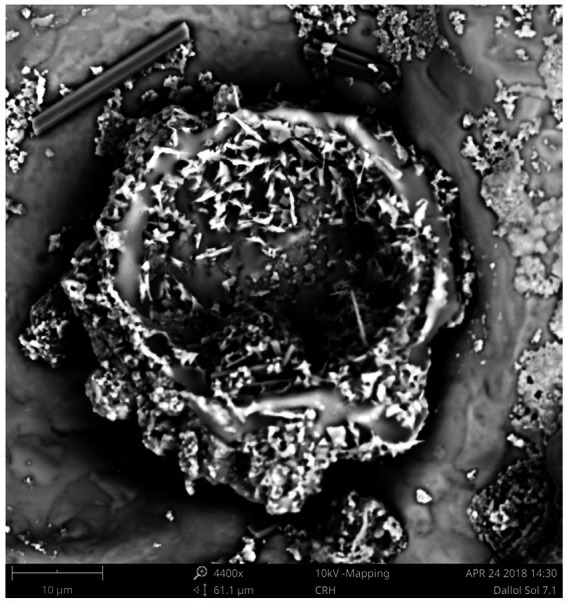
SEM pictures of a residue of the brine of the Central green pool (Outcrop zone) showing most likely the result of a microbial fossilization process or the shapes of abiotic biomorphs.

Although our SEM-analyses occasionally showed microbial like shapes, even of samples of the outcrop zone (Figure 8), the EDX element analyses of the microbial like shapes rarely confirmed the presence of biological related elements like carbon, nitrogen, or phosphorus. The microbial shapes had almost always the same composition as the attached mineral. Therefore, we assumed that these observations were the result of biomorphs, abiotic structures resembling biological shapes, or the result of a natural microbial fossilization process. As opposed to microbial degradation, microbial fossilization can occur under special circumstances. Typically in environments were rapid mineral precipitation is likely to happen. The hyper saline halo-volcanic Dallol complex is such an environment where the microbial fossilization process is likely to occur (Walter and Allwood, 2005).

A 2019 study of Gómez suggested the existence of ultra-small microorganisms in the poly extreme waterbodies of the Dallol outcrop zone (Gomez et al., 2019). The conclusions were essentially based on the results of a combination of morphological and molecular analyses. Their results were not supported with chemical or biochemical analyses of metabolites that could indicate an active biology. In contrast of the 2019 study of Gómez, our microbiological survey did not detect the existence of an active archaeal life in the poly extreme waterbodies of the outcrop zone. Nor any positive isolation or cultivation result. Also, Belilla et al. (2019) published their work: Hyperdiverse archaea near life limits at the polyextreme geothermal Dallol area. Their extended multidisciplinary microbiological survey indicated the absence of any active microbiological life in the poly extreme waterbodies of the outcrop zone. Their observations and analyses are in agreement with our analyses. Although we succeeded to extract DNA from certain waterbodies of the outcrop zone, none of the OTU’s made sense in the context of an extreme acid and saline environment. Quasi all OTU’s could be directly related to human or animal microbiomes. We considered this as normal as during our sampling of the Dallol waterbodies, occasionally groups of dragonflies flew over the site, descended to consume the poisonous and deadly liquid of the waterbody, after which some disappeared and sank into the brine solution. We also discovered numerous dead flying eukaryotic animals in the close proximity on the shores of the waterbodies (cfr. Supplementary Figure S8).

## 4. Conclusion

All our measurements of the 10 investigated hyper saline lakes of the halo-volcanic Dallol complex, clearly indicated the exceptional extreme geological and physico-chemical nature of the region. Although the Dallol region was relatively small, it could still easily be divided into three smaller and very different subzones. This partitioning became also evident by the observed differences in geochemistry, mineralogy, and the different origins of the water of the brines. Our geological, physico-chemical, mineralogical, and microbiology analyses of samples of the halo-volcanic Dallol complex are in contrast with the 2019 Gómez study which suggests the presence of ultra-small microorganisms in the poly extreme waterbodies of the Dallol outcrop zone (Gomez et al., 2019). However, our results fully support the conclusions of Belilla et al. (2019). We also have to conclude that kosmo-chaotropicity and water activity are the two most important physico-chemical constraints for life. When it is proven that the kosmo-chaotropicity value of a geological zone exceeds 87.3 kJ.kg^−1^ or its A_w_ value is below 0.611, we should conclude that such zone is factual lifeless. Besides, the combination of extreme low pH and high salinity, even if this salinity is caused by the kosmotropic salt sodium chloride, is deleterious for any biological life form. However, our survey could identify several known halophilic archaea, *Halobaculum, Halobellus, Haloplanus, Natronarchaeum, Halomicroarcula, Halorientalis,* canditatus *Nanosalina*, and *Limimonas*, and one salt loving bacterial genus, *Salinibacter*, in the waterbodies that were fully saturated with sodium chloride but only mildly acidic. Although, no one can ever provide the prove of the total absence of life, our extended multidisciplinary survey strongly indicates that the halo-volcanic Dallol complex contains real lifeless geological zones, even zones in which waterbodies are present. In geological zones, so extreme that even chemical molecules have to fight to earn their essential crystal water, no biological life can exist. Our results and conclusions might be beneficial to more precisely consider areas of celestial objects as worthy or unworthy to be investigated in the frame of discovering extraterrestrial life.

## Data availability statement

The datasets presented in this study can be found in online repositories. The names of the repository/repositories and accession number(s) can be found at: NCBI—PRJNA927119.

## Author contributions

HM wrote the manuscript. MC contributed to the fieldwork. HM, CS, and AP performed the laboratory experimental works and sample preparations of samples that were outsourced. NL managed the microbiology research team of SCK CEN, made the fieldwork and laboratory analyses possible, and supported the whole project. All authors contributed to the article and approved the submitted version.

## Funding

This work follows field work conducted in January 2018 at the Danakil Planetary Field Analog Site (TA1-5) of Europlanet 2020 RI, which has received funding from the Horizon 2020 research and innovation program of European Union under grant agreement No 654208.

## Conflict of interest

The authors declare that the research was conducted in the absence of any commercial or financial relationships that could be construed as a potential conflict of interest.

## Publisher’s note

All claims expressed in this article are solely those of the authors and do not necessarily represent those of their affiliated organizations, or those of the publisher, the editors and the reviewers. Any product that may be evaluated in this article, or claim that may be made by its manufacturer, is not guaranteed or endorsed by the publisher.
