# Supplementary material for: The waterbodies of the halo-volcanic Dallol complex: earth analogs to guide us, where to look for life in the universe
Source: Front Microbiol. 2023 Jul 14;14:1134760. doi: 10.3389/fmicb.2023.1134760 (PMC10382021; doi:10.3389/fmicb.2023.1134760)
Supplement: Supplementary file 1 [file Data_Sheet_1.PDF]

Supplementary material

The waterbodies of the halo-volcanic Dallol complex: Earth analogues to guide us, where to look for life in the universe.

Hugo Moors[1*], Mieke De Craen[2,3], Carla Smolders[1], Ann Provoost[1] and Natalie Leys[1]

[1] Microbiology Unit, Belgian Nuclear Research Centre (SCK CEN), Nuclear Medical Applications institute (NMA), Boeretang 200, Mol, Belgium

[2] Research and Development Disposal, Belgian Nuclear Research Centre (SCK CEN), Waste and Disposal (W&D), Boeretang 200, Mol, Belgium

[3] current affiliation: European Underground Research Infrastructure for Disposal of Nuclear Waste in Clay Environment, EIG EURIDICE, Boeretang 200, Mol, Belgium

*** Correspondence:**
Hugo Moors
hugo.moors@sckcen.be

Keywords: Dallol, habitability, chaotropicity, water activity, salinity

Contents

1 List of all samples taken from the halo-volcanic Dallol complex....2
2 Additional Physico-chemical data....3
3 Cultivation protocols and media....4
3.1 SAM medium: Simulated Asale Medium....4
3.2 DSMZ 936 medium: Salinibacter ruber medium....4
3.3 DSMZ 97 medium: Halobacterium medium....5
3.4 DSMZ 88 medium: Sulfolobus medium....5
3.5 DSMZ 1336 medium: Sehgal and Gibons medium....5
3.6 A63 medium: Medium to cultivate sulphate reducing microbes....6
4 Additional SEM analyses....6
4.1 The outcrop zone....7
4.2 The south-southeast base zone....8
4.3 The flat south plane zone....9
5 Supplementary microbiology information....10
5.1 Rarefraction analysis....12

1 List of all samples taken from the halo-volcanic Dallol complex

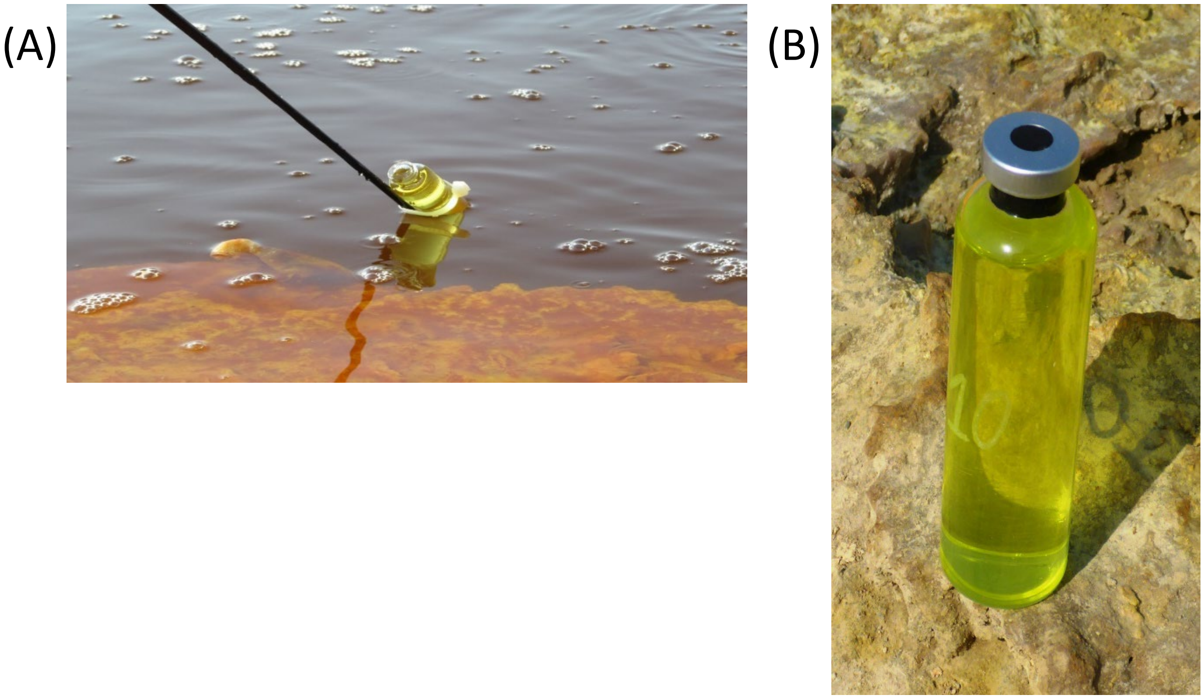

Figure S1: (A) Visualization of the technique used to collect liquid brine samples. (B) To assure long term sample integrity and representativeness, collection was performed in 50 ml glass bottles,

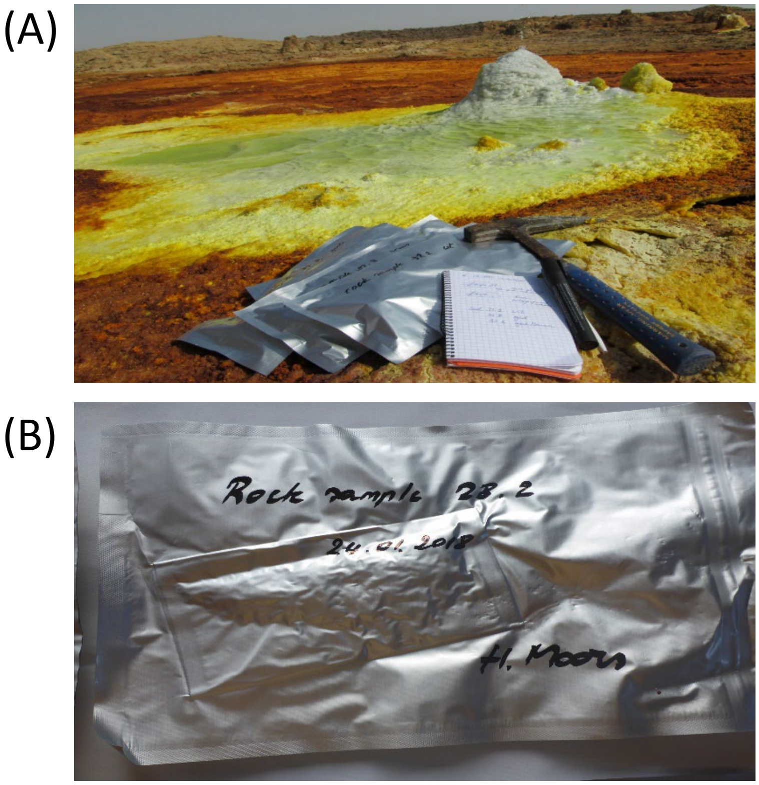

thereby, avoiding the presence of any interfering gaseous headspace. The sample bottles were on the spot immediately hermetically sealed with a 13 mm tick butyl rubber septum.

Figure S2: Solid and sediment samples were hand picked, wearing sterile surgical gloves. To assure long term sample integrity and representativeness, samples were put into Al-PE bags (A), which were made vacuum and heat sealed (B) upon arrival at the base campThe excel file: "Supp_material_Table_1.xlsx" contains a list of all samples, liquid as well as solid taken from the halo-volcanic Dallol complex.

2 Additional Physico-chemical data

Figure S3 illustrate the weight based procedure for determining the Total Disolved Salt, TDS) measurement

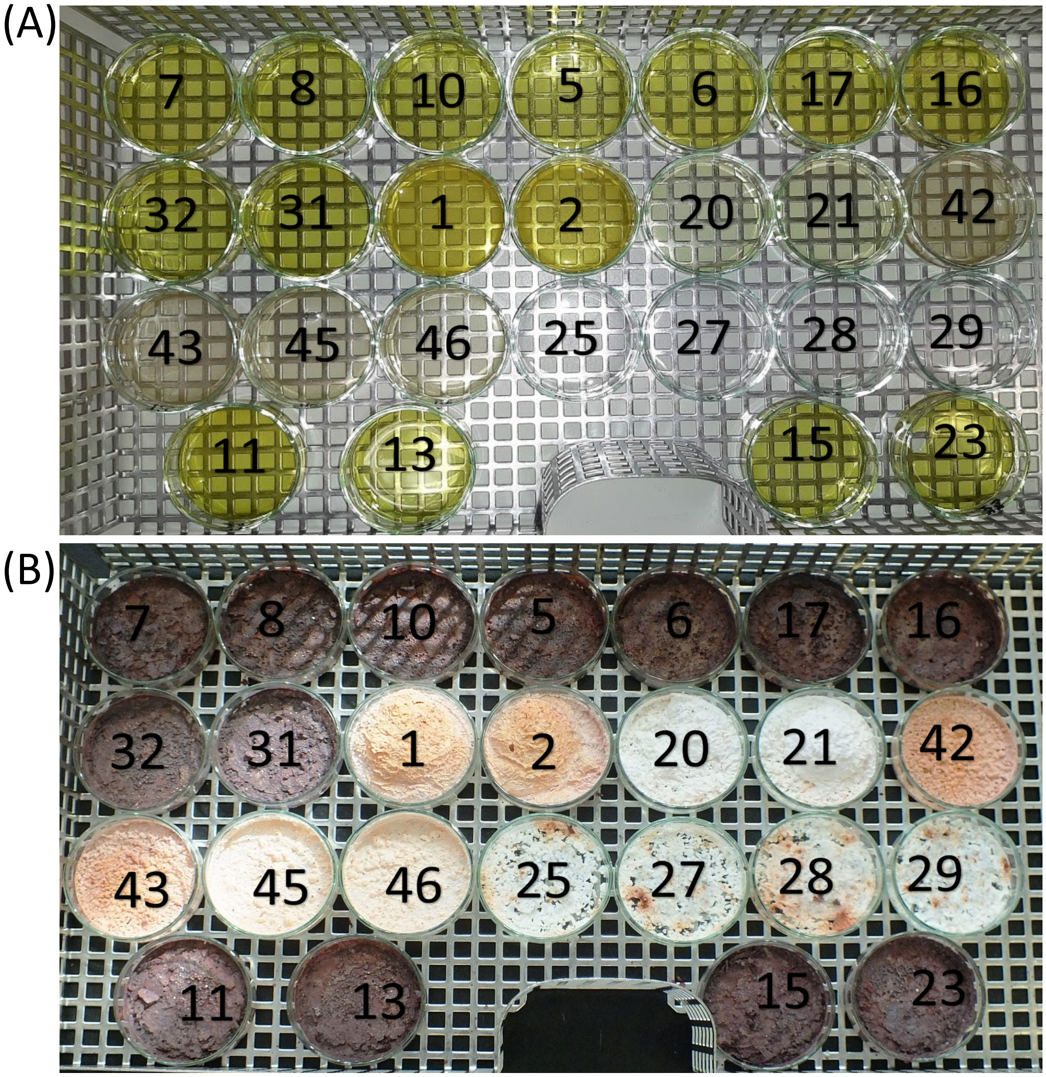

Figure S3: Gravimetric determination of the Total Dissolved Salts (TDS) content. (A) Before drying and (B) after drying at 240°C for a period of about 24 hours.

A complete overview as table with conditional formatting can be consulted in "Supp_material_2.xlsx"

3 Cultivation protocols and media

3.1 SAM medium: Simulated Asale Medium

SAM medium is specifically designed for the cultivation of halophilic microorganisms potentially thriving in waterbodies of the flat south zone of the halo-colcanic Dallol complex. In parallel, it combines some cultivation promoting components found in the common R2A medium. The latter is generally used to cultivate oligotrophic microorganisms. SAM medium primarily contains sodium and potassium chloride to generate the appropriate high salinity of the natural environments of halophilic organisms. Beside, sodium and potassium, also divalent cations calcium and magnesium are present. The main anion is chloride together with low dosed bromide. Traces of bicarbonate and sulphate may trigger autotrophy and/or provide an appropriate electron acceptor and essential sulphur source. The necessary amount of phosphate is also present under the form of a dipotassium salt. SAM medium offers a huge diversity of carbon sources in low concentrations to promote oligotrophic microbes. A low concentration of a nitrogen and iron source is added under the form of iron(III)ammonium citrate, in which the citrate is used to stabilize the present carbon sources.

Preparation of one liter of SAM medium

Proteose peptone (Merck)	0.5 g
Casein hydrolysate (Merck)	0.5 g
Yeast extract	0.5 g
Sodium chloride	175.0 g
Potassium chloride	5.6 g
Calcium chloride dihydrate	10.0 g
$MgSO_4 \cdot 7\ H_2O$	5.0 g
$K_2HPO_4 \cdot 3H_2O$	0.1 g
Potassium bromide	0.5 g
Sodium bicarbonate	0.25 g
Iron(III)ammonium citrate	0.005 g

Adjust pH to 5.0±1 @ 25 °C

• Autoclave in a vented open bottle at 121°C

Initiating the growth of microbial cultures was performed as follows:

Sterile glass bottles were each filled, under the protection of a laminar flow cabinet (LAF), with 10 ml of the appropriate medium. Each bottle was hermitically closed with a thick sterile chloro-butyl septum and aluminum crimp cap. Each different medium was inoculated with 0.5 ml (5%) of the original *in situ* collected liquid samples. OD_{600} measurements were recorded immediately after inoculation as a reference culture specific starting point. Incubation was performed at 37°C to mimic *in situ* temperature conditions.

3.2 DSMZ 936 medium: Salinibacter ruber medium

NaCl 195.000 g

$MgCl_2$ x 6 H_2O 34.600 g

$MgSO_4$ x 7 H_2O 49.500 g

$CaCl_2$ x 2 H_2O 1.250 g

KCl 5.000 g

$NaHCO_3$ 0.250 g
NaBr 0.625 g
Yeast extract 1.000 g
Distilled water 1000.000 ml
Final pH 7.2. Liquid media may be solidified with 20 g/l agar.

3.3 DSMZ 97 medium: Halobacterium medium

Casamino acids 7.50 g
Yeast extract 10.00 g
Na3-citrate 3.00 g
KCl 2.00 g
$MgSO_4$ x 7 H_2O 20.00 g
$FeSO_4$ x 7 H_2O 0.05 g
$MnSO_4$ x H_2O 0.20 mg
NaCl 250.00 g
Agar 20.00 g
Distilled water 1000.00 ml
Adjust pH to 7.4. Add the agar after dissolving all ingredients in the water and adjustment of pH.

3.4 DSMZ 88 medium: Sulfolobus medium

$(NH_4)_2SO_4$ 1.30 g
KH_2PO_4 0.28 g
$MgSO_4$ x 7 H_2O 0.25 g
$CaCl_2$ x 2 H_2O 0.07 g
FeCl3 x 6 H_2O 0.02 g
Allen’s trace element solution (see below) 10.00 ml
Yeast extract (OXOID) 1.00 g
Distilled water 1000.00 ml
Dissolve ingredients (except yeast extract or other substrates), adjust pH of the salt solution at room temperature to 2.0 using 10 N H2SO4 and autoclave. Yeast extract and other organic substrates are sterilized separately by autoclaving of a 10% (w/v) stock solution at neutral pH.
Allen’s trace element solution:
$MnCl_2$ x 4 H_2O 180.00 mg
$Na_2B_4O_7$ x 10 H_2O 450.00 mg
$ZnSO_4$ x 7 H_2O 22.00 mg
$CuCl_2$ x 2 H_2O 5.00 mg
Na_2MoO_4 x 2 H_2O 3.00 mg
$VOSO_4$ x 2 H_2O 3.00 mg
$CoSO_4$ 1.00 mg
Distilled water 1000.00 ml
Adjust pH of final solution to 2 with 1 N HCl.

3.5 DSMZ 1336 medium: Sehgal and Gibons medium

NaCl 40.0 g
KCl 2.0 g
$MgSO_4$ x 7 H_2O 20.0 g

Yeast extract 10.0 g
Tryptone 5.0 g
Trisodium citrate dehydrate 3.0 g
Casein hydrolysate 5.0 g
Dest. Water 1000.0 ml
Adjust pH to 7.2-7.4.

3.6 A63 medium: Medium to cultivate sulphate reducing microbes

Adapted medium 63 (A63-medium) is a versatile medium to cultivate Sulphate reducing prokaryotes (SRP). It uses yeast extract and lactate as carbon sources and several sulphate salts as electron acceptor providers. In contrast to the normal used sodium thioglycolate, L-Cysteïne hydrochloride and ascorbic acid are added as the redox controlling and lowering ingredients. Iron sulphate is added to protect the SRP's from being inhibited in their growth by their own toxic sulphide formation. The blackening of the medium caused by iron sulphide precipitations (=sulphide removal mechanism) allows easy control of SRP growth. Resazurin is added as a reliable indicator of unwanted oxidation of the A63-medium.

Preparation of one liter of A63-medium

Three solutions, A, B and C have to be made mixed and finally sterilized by autoclaving.

Solution A:

K_2HPO_4	0.5 g
NH_4Cl	1.0 g
Na_2SO_4	1.0 g
$CaCl_2 \cdot 2H_2O$	0.1 g
$MgSO_4 \cdot 7H_2O$	2.0 g
DL-Na-lactate	2.0 g
Yeast extract	1.0 g
Resazurin	1.0 mg
Distilled water	980.0 ml

Solution B:

$FeSO_4 \cdot 7H_2O$	0.5 g
Distilled water	10.0 ml

Solution C:

L-Cysteïne hydrochloride	0.5 g
Ascorbic acid	0.1 g
Distilled water	10 ml

Dissolve the ingredients of each solution in the appropriate quantities of water. Bring solution A to the boil for a few minutes, then cool to room temperature while gassing with oxygen-free N_2 gas or under the atmospheric protection of an anaerobic glove box. Add solutions B and C (may also be stored under the atmospheric protection of an anaerobic glove box), adjust pH to 7.8 with NaOH (5M), and distribute in anaerobic septum vials or hungate tubes. During distribution continuously swirl the medium to keep the grey precipitate equally suspended.
Autoclave filled septa or hungate tubes for 15 min at 121°C.

4 Additional SEM analyses

4.1 The outcrop zone

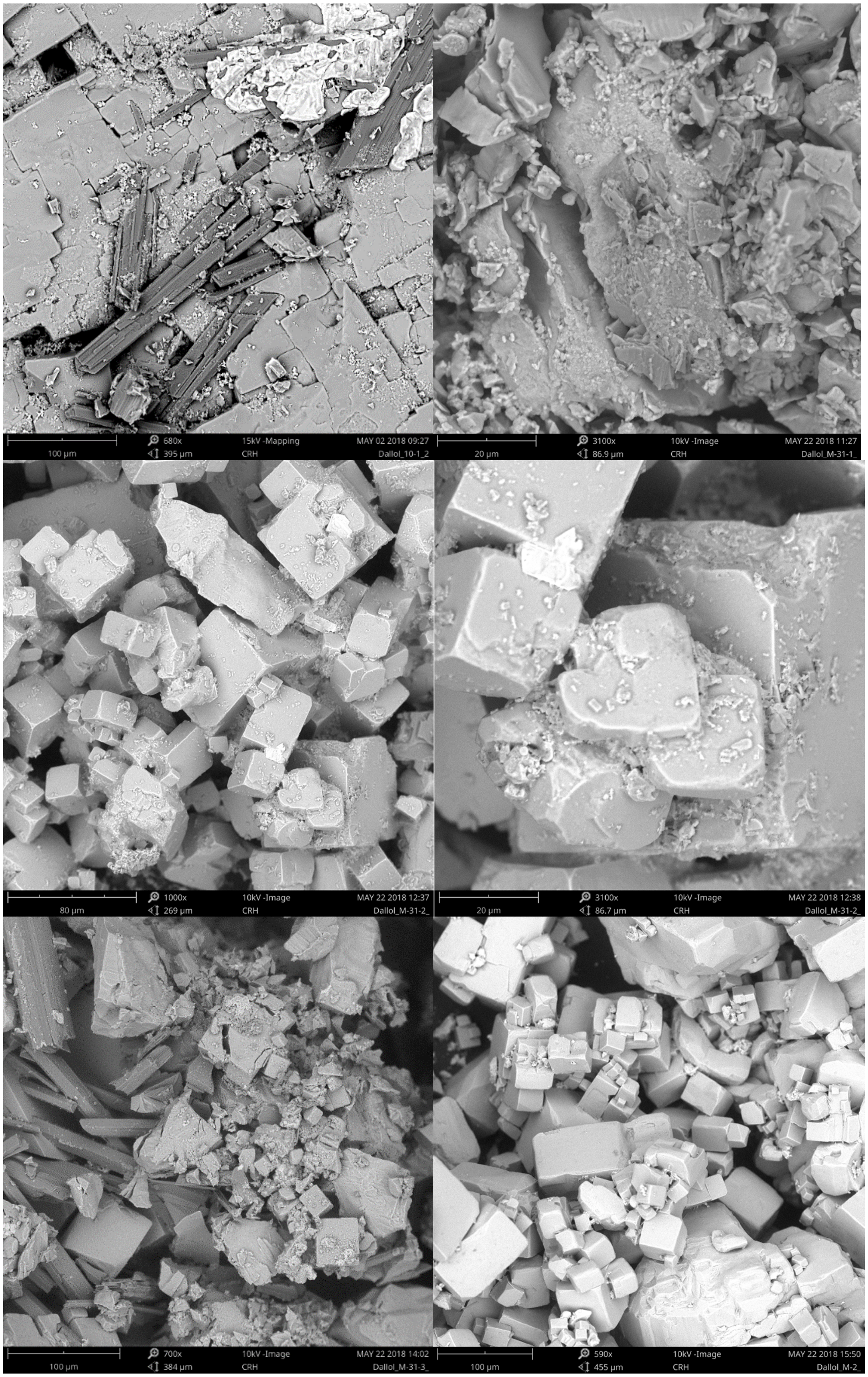

Figure S4: Typical SEM images of sediment materials/minerals of the waterbodies of the outcrop zone

4.2 The south-southeast base zone

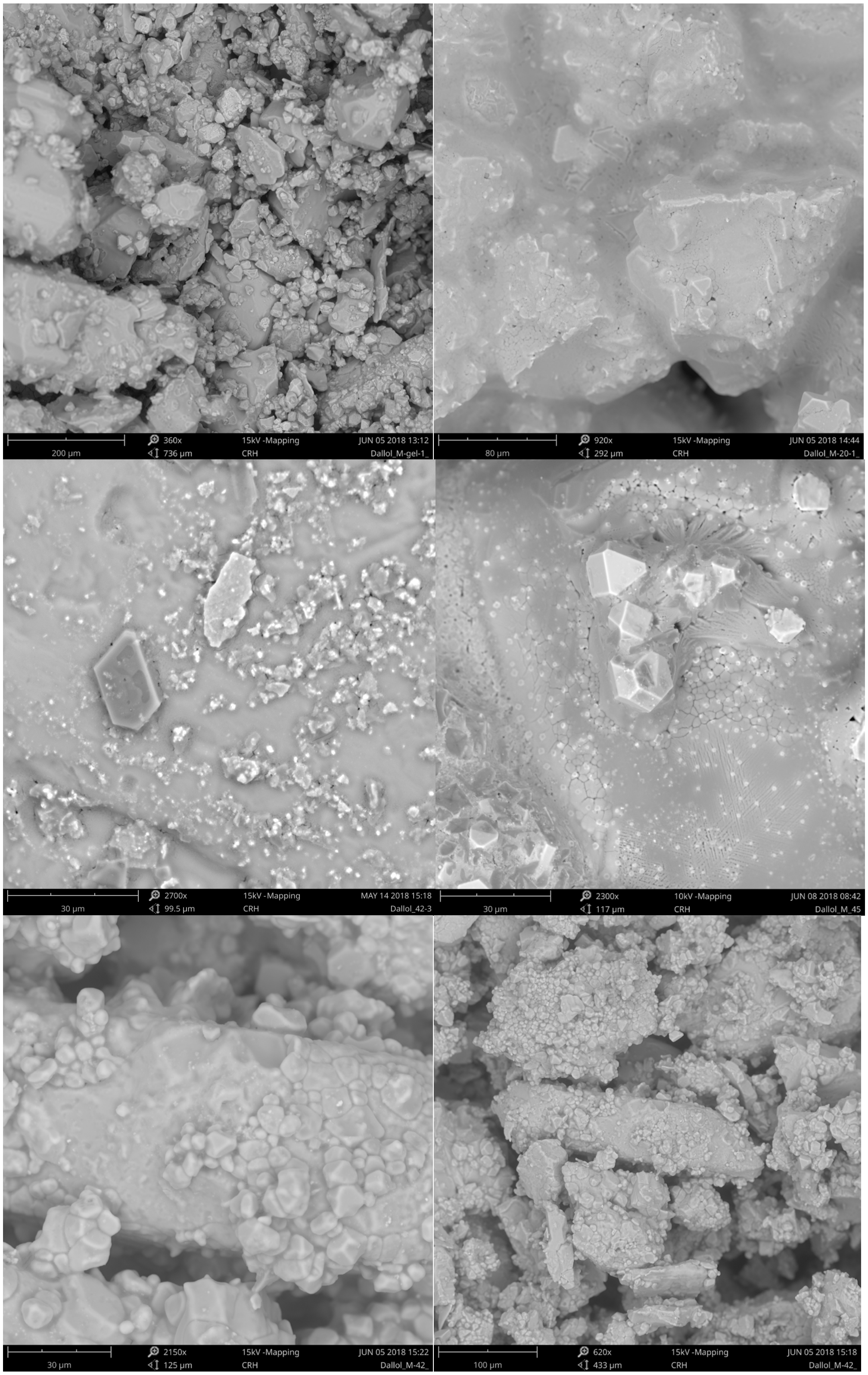

Figure S5: Typical SEM analyses of sediments/minerals of the waterbodies of the south-southeast base zone

4.3 The flat south plane zone

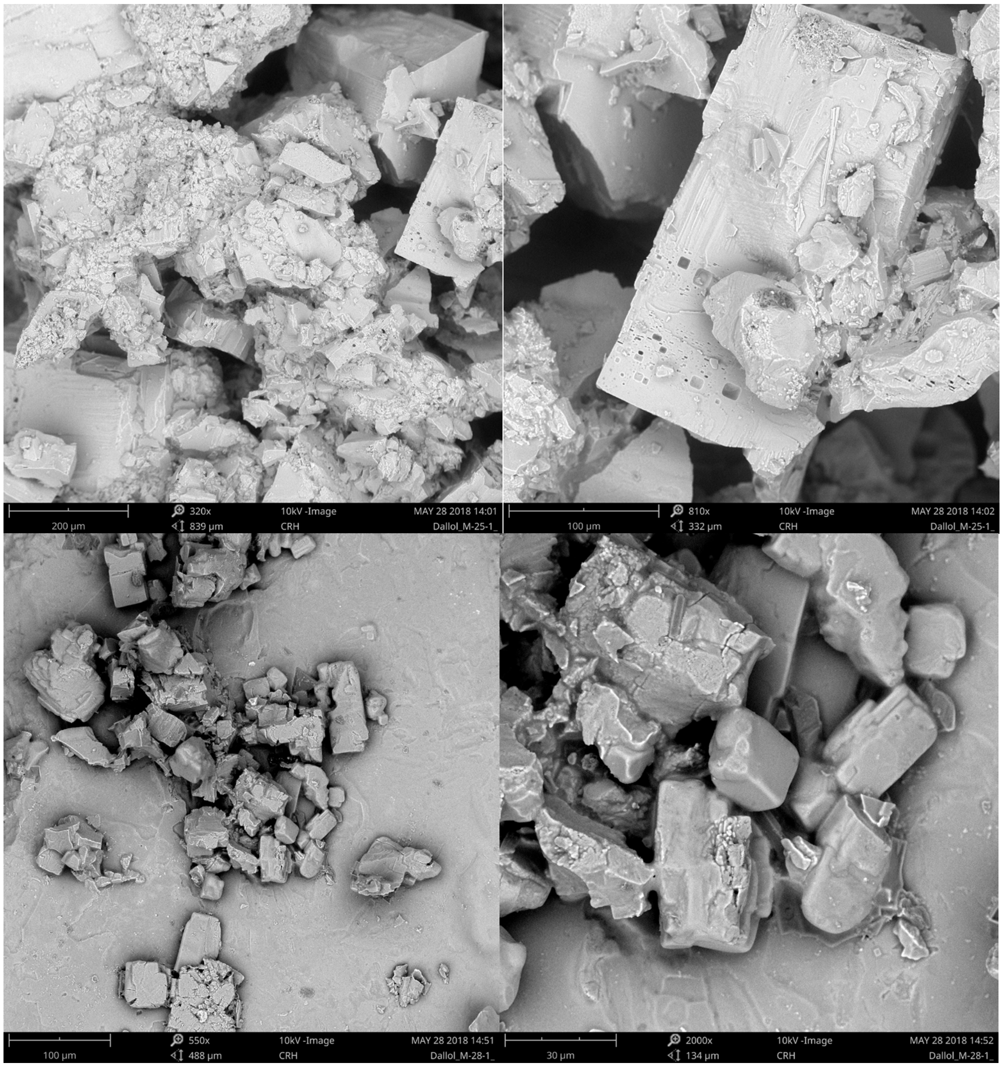

Figure S6: Typical SEM analyses of the sediments/minerals of waterbodies of the flat south plane zone

5 Supplementary microbiology information

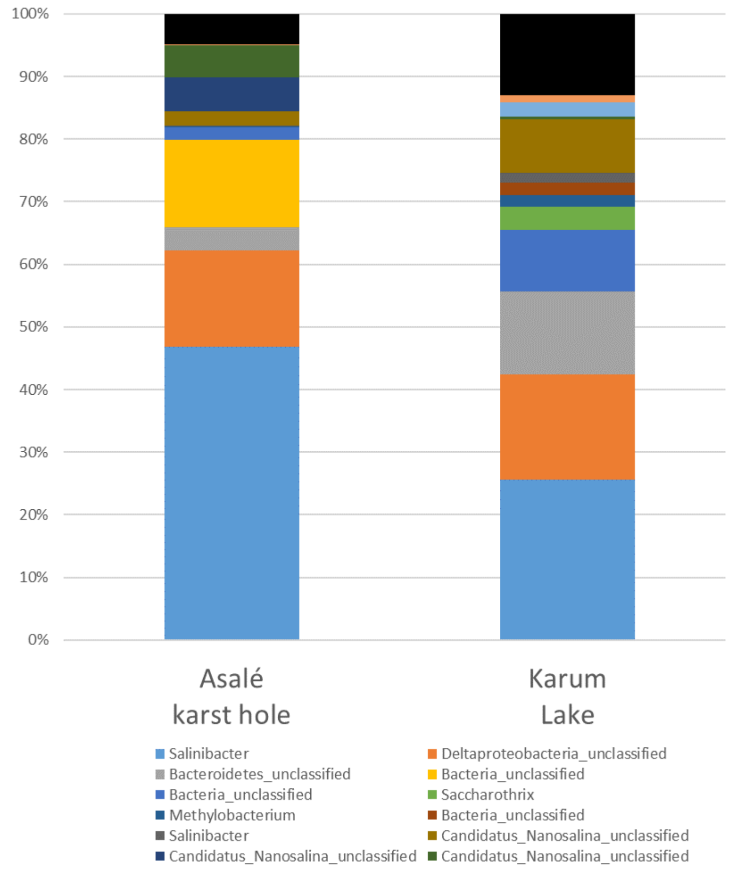

Figure S7: Taxonomy and relative occurence of the most dominant most likely indigenous microbial species detected in the brines of the Asale karst hole and the Karum lake

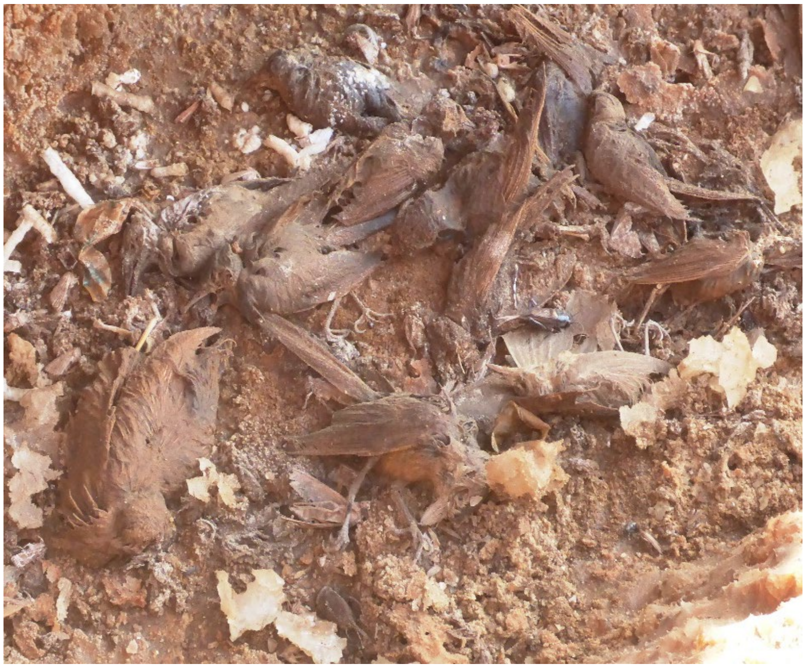

Figure S8: Picture taken in the close vicinity of the shores of the Gaet'ale Pond showing numerous dead flying animals that were killed as a result of frinking the toxic brine solution or by suffocation by the presence of high concentrations of toxic volcanic gases.

5.1 Rarefraction analysis

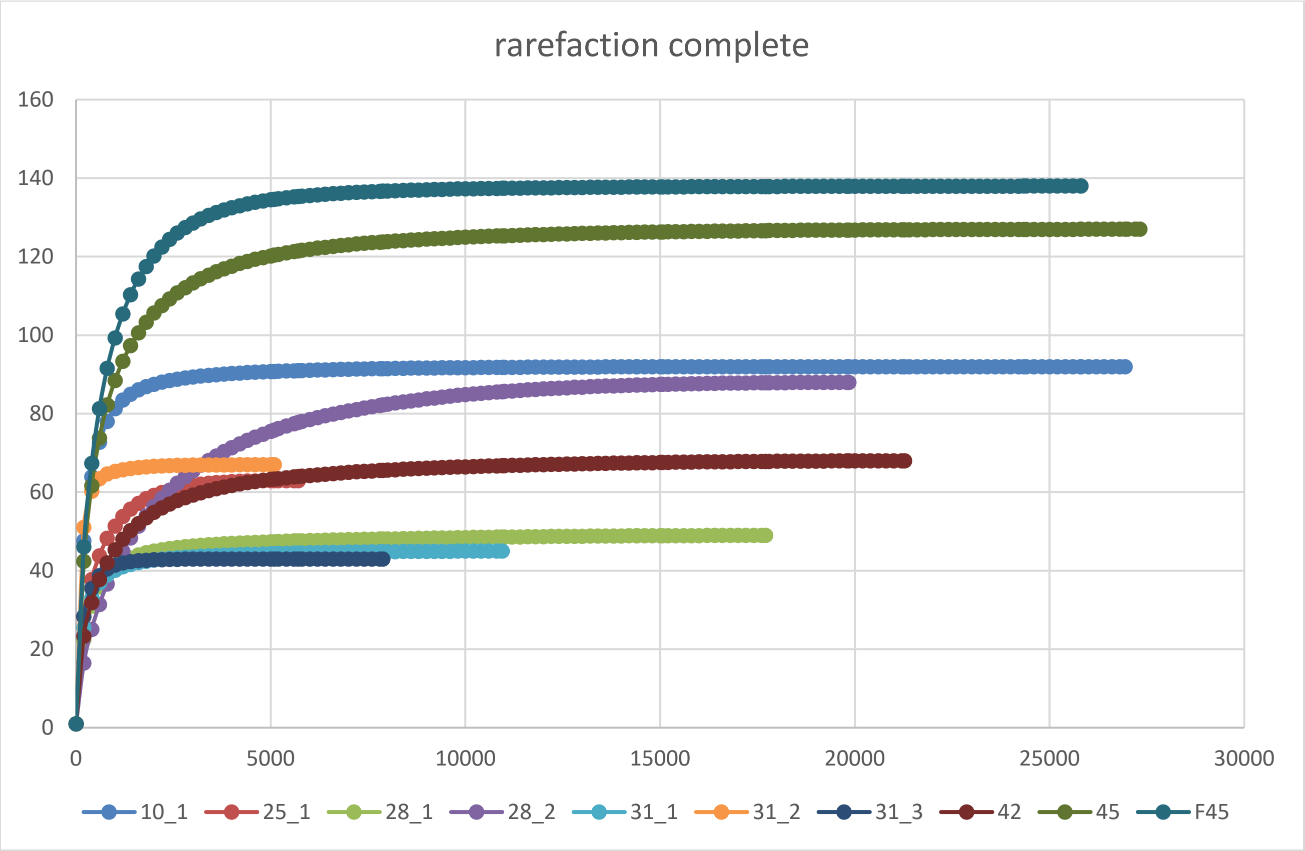

Complete rarefraction curve of the 16S rDNA community analyses performed on the DNA extracted from the original Dallol samples. This figure confirms that the results of the community analyses can be considered as sufficiently accurate.

A comprehensive summary of our 16S rDNA analyses performed on the DNA that was extracted from 10 samples from the brine solutions of the halo-volcanic Dallol complex can be consulted in the excel file: Supp_material_Table_3.xlsx. This excel file contains a sheet with a sample summary, the complete list of OTU's discovered after treating the raw data with our bioinformatics OCToPUS pipeline and a complete list of the consensus DNA sequences.

An additional 16S rDNA analysis, using primers that were better adapted for the discovery of Archaea, can be consulted in the excel file: Supp_material_Table_4.xlsx. This analysis was only performed on the two brines of the flat south plane zone. The zone that most likely contained indigenous living microbes.
